# scExplorer: a comprehensive web server for single-cell RNA sequencing data analysis

**DOI:** 10.1093/bioadv/vbaf273

**Published:** 2025-11-03

**Authors:** Sergio Hernández-Galaz, Andrés Hernández-Olivera, Felipe Villanelo, Alvaro Lladser, Alberto J M Martin

**Affiliations:** Laboratorio de Redes Biológicas, Fundación Ciencia and Vida, Huechuraba, Santiago, 8580702, Chile; Laboratorio de Inmunooncología, Fundación Ciencia and Vida, Huechuraba, Santiago, 8580702, Chile; Centro de Investigación e Innovación en Cáncer, Fundación Arturo López Pérez, Providencia, Santiago, 7500691, Chile; Laboratorio de Inmunooncología, Fundación Ciencia and Vida, Huechuraba, Santiago, 8580702, Chile; Facultad de Ingeniería, Universidad San Sebastián, Santiago, 8420524, Chile; Laboratorio de Inmunooncología, Fundación Ciencia and Vida, Huechuraba, Santiago, 8580702, Chile; Facultad de Medicina, Universidad San Sebastián, Providencia, Santiago, 7510157, Chile; Laboratorio de Redes Biológicas, Fundación Ciencia and Vida, Huechuraba, Santiago, 8580702, Chile; Facultad de Ingeniería, Universidad San Sebastián, Santiago, 8420524, Chile

## Abstract

**Summary:**

Computational analysis of single-cell RNA sequencing (scRNA-seq) data presents significant barriers for researchers lacking programming expertise, particularly for multi-dataset integration, scalable job management, and reproducible workflows. We developed scExplorer, a web-based platform that addresses these limitations through three key innovations: Comprehensive batch correction using four state-of-the-art algorithms (ComBat, Scanorama, BBKNN, and Harmony), SLURM-based job scheduling with pause/resume functionality for large-scale analyses, and automated generation of publication-ready reports with exportable configuration files ensuring complete reproducibility. The platform’s modular Docker architecture supports both standalone and client–server deployments, enabling analysis of datasets ranging from thousands to hundreds of thousands of cells. An openly documented REST API clarifies how the interface orchestrates analyses and supports transparent operation. scExplorer eliminates the technical barriers that prevent non-computational researchers from performing rigorous scRNA-seq analysis while maintaining the transparency and reproducibility standards required for collaborative research.

**Availability and implementation:**

https://apps.cienciavida.org/scexplorer/.

## 1 Introduction

Advances in next-generation sequencing technologies have enabled the development of single-cell RNA sequencing (scRNA-seq), a powerful approach that captures gene expression profiles at individual cell resolution. Since the introduction of this technique in 2009, scRNA-seq has facilitated the identification of novel cellular markers and provided insights into continuous processes critical to developmental biology and various physiological phenomena (Aldridge and Teichmann 2020, Fan *et al.* 2020, Rood *et al.* 2022). The field has experienced remarkable growth, as evidenced by the exponential increase in publications indexed in PubMed, expanding from under 100 papers in 2009 to ∼5000 publications by early 2024. This dramatic surge in scientific output reflects the technology’s increasing adoption and importance across biological disciplines.

In parallel to this publication trend, there has been substantial growth in publicly available scRNA-seq datasets. Data repositories such as the Gene Expression Omnibus (GEO) (Barrett *et al.* 2013) and the Human Cell Atlas Data Portal (Regev *et al.* 2017) have seen a significant expansion in their data collections. For instance, the Single Cell Expression Atlas (Papatheodorou *et al.* 2020) hosts thousands of experiments comprising millions of cells across diverse species and tissue types, making it an invaluable and steadily growing resource for the scientific community.

Building on the increasing relevance of scRNA-seq, several tools have been developed to analyse scRNA-seq data systematically. Prominent among these are Seurat in R (Butler *et al.* 2018) and Scanpy in Python (Wolf *et al.* 2018). However, the requirement for programming skills remains a major obstacle, preventing many scientists from effectively interpreting their data. While several web-based platforms have emerged to address this, many present new hurdles for rigorous, collaborative research, including opaque analysis pipelines that hinder reproducibility, the inability to manage long-running jobs on shared hardware, and inflexible methods for integrating complex, multi-batch datasets.

We developed scExplorer to address these specific technical barriers while maintaining the accessibility advantages of web-based platforms. Our approach combines comprehensive batch correction algorithms, scalable SLURM-based job management, and automated reproducibility features within a code-free interface. This enables researchers to perform rigorous, publication-quality scRNA-seq analysis regardless of their computational background, while ensuring that results can be precisely replicated and extended by collaborators.

## 2 Methods

scExplorer provides an integrated pipeline for scRNA-seq analysis, from raw data import through differential expression analysis and visualization. The platform accepts multiple input formats (.h5ad,.h5,.rds, and 10× Cell Ranger outputs) to accommodate diverse data generation workflows.

### 2.1 Quality control and preprocessing

We implemented standard quality control procedures to ensure high-quality input for downstream analysis. Low-quality cells and uninformative genes are filtered to reduce technical noise and improve result reliability. The pipeline removes cells with insufficient gene expression and filters genes expressed in very few cells, preventing statistical artefacts in downstream analyses.

Cells exhibiting excessive mitochondrial gene expression—often indicating cellular stress or damage—are excluded by default. We integrated Scrublet (Wolock *et al.* 2019) for doublet detection, preventing merged cells from being misinterpreted as distinct cell populations and preserving single-cell resolution.

Following quality control, we normalize raw counts using standard log-normalization: counts are scaled to 10 000 per cell, then log-transformed with a pseudocount of one (log1*P*). This approach maintains consistency with established Seurat and Scanpy workflows.

### 2.2 Highly variable gene selection

To capture the most informative biological variation, we identify highly variable genes (HVGs) using either Seurat or Cell Ranger methods. The Seurat approach stabilizes variance across datasets, particularly beneficial for multi-batch analyses. The Cell Ranger method optimizes performance for 10× genomics-generated datasets.

### 2.3 Dimensionality reduction and clustering

We perform dimensionality reduction using UMAP (Becht *et al.* 2019) followed by cell clustering with the Leiden algorithm (Traag *et al.* 2019), which improves upon the established Louvain method by ensuring well-connected communities. The resolution parameter controls clustering granularity, allowing exploration of both broad cell populations and fine-grained subpopulations. To guide optimal clustering resolution selection, we integrated Clustree (Zappia and Oshlack 2018), which visualizes how cluster structure changes across different resolution parameters. This helps users make informed, objective decisions about appropriate granularity for their specific dataset, reducing subjective bias in cell type identification.

Users can assign biologically meaningful labels to clusters through an interactive interface. These annotations are stored in the data object and propagate automatically to all visualizations and exported files (.h5ad,.rds), ensuring consistent labelling across sessions and collaborators.

### 2.4 Differential expression analysis

We identify cluster-specific marker genes using either the Wilcoxon rank-sum test or Student’s *t*-test. The Wilcoxon test, being non-parametric, handles the non-normal distributions typical of scRNA-seq data, while the t-test provides higher statistical power when normality assumptions are met. The system can automatically identify top-ranking genes for each cluster or evaluate user-specified gene lists, supporting both exploratory and hypothesis-driven analyses.

### 2.5 Visualization and export

Interactive visualizations built with Plotly (Plotly Technologies Inc. 2015) support extensive customization including colour palettes, font families and sizes, axis labels, legend placement, and export resolution. A Theme Manager provides accessibility-focused presets (colour-blind friendly, high-contrast) to improve inclusivity. For datasets exceeding ∼100 000–200 000 cells, plots automatically switch to rasterized rendering to preserve interactivity while maintaining visual fidelity; documentation also provides guidance on subset-based strategies for atlas-scale studies. All plots export in both SVG and PNG formats for publication use. A high-resolution heatmap module generates cell-by-gene or cluster-by-gene summaries with optional z-scoring and hierarchical clustering. Gene selection can be based on top markers or custom lists, with full Theme Manager integration for consistent styling. All analysis results export in both Python and R formats, facilitating integration with existing computational workflows.

### 2.6 System architecture

scExplorer uses a modular Docker-based architecture that isolates components including the frontend (Node.js/Express), backend (FastAPI), analysis modules (Scanpy and Seurat), and job scheduling (SLURM). This design ensures scalability, simplifies maintenance, and enables efficient resource management for both small and large datasets. Each analysis receives a unique UUID displayed in the interface and included in email notifications. Users can resume analyses at any time using this identifier, which restores saved parameters and enables continuation from the last consistent checkpoint. UUIDs are preserved in automated reports and exported configurations for precise provenance tracking.

Users interact with a lightweight web frontend that communicates with a remote HPC backend. SLURM integration provides job queuing and email notifications, enabling resource-intensive analyses on shared infrastructure.

### 2.7 Software installation and deployment

We provide step-by-step installation guides in the public repository, including Docker Compose deployment, environment configuration, and SLURM integration. Installation has been validated on Linux, macOS, and Windows. Administrators can configure data directories, per-file size limits, and email notifications. The upload interface explicitly lists accepted file formats (.h5ad,.h5,.rds, and 10× Cell Ranger directories) and the configurable maximum file size directly adjacent to the upload control and via tooltips, improving user guidance.

Client–server deployment is explicitly supported: the frontend can run locally while jobs are submitted to a remote SLURM-backed backend. The repository details the necessary environment variables, URL configuration, and network setup to enable this mode in shared computing environments.

**Figure 1.**
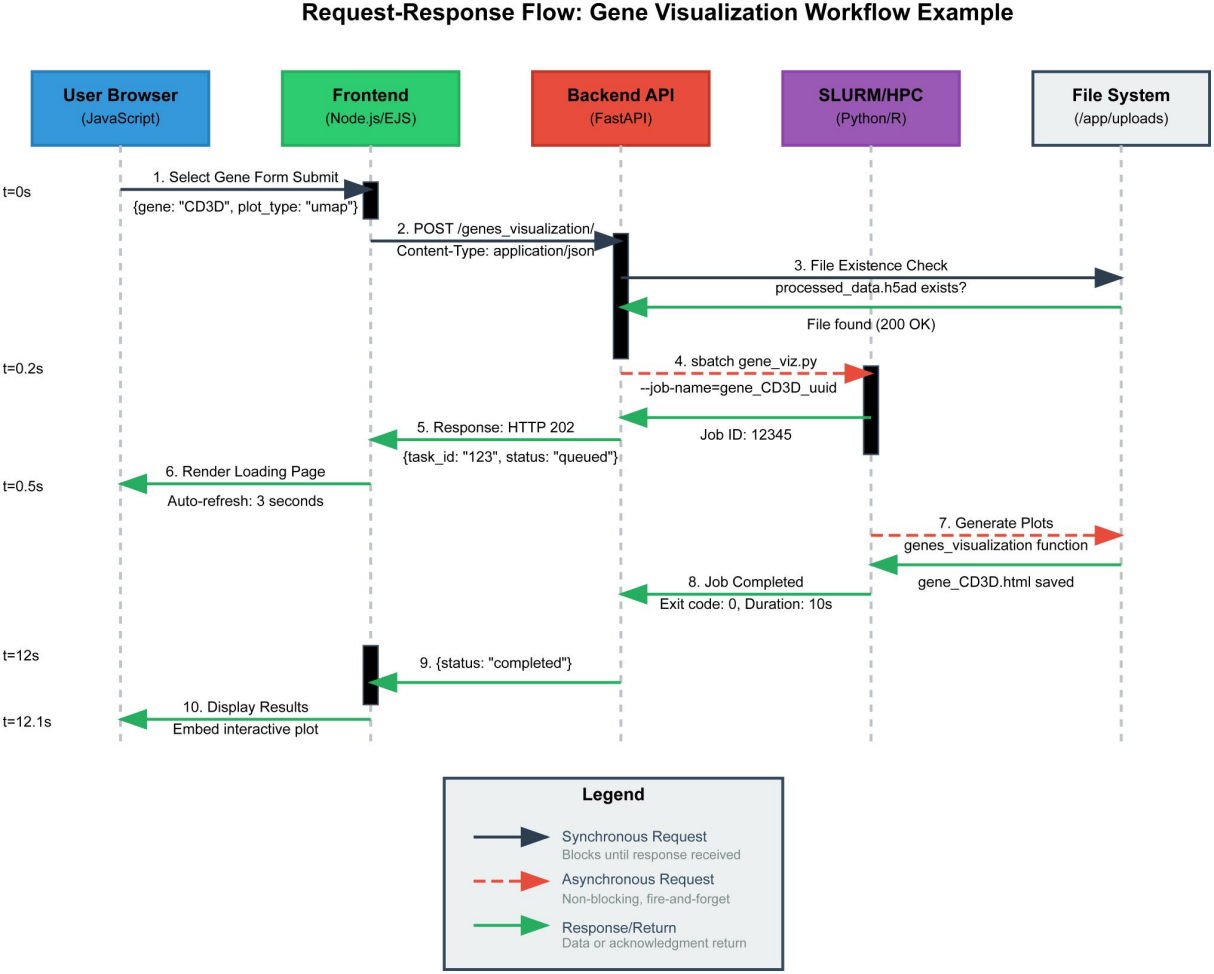
Request-response flow for gene visualization in scExplorer architecture. The diagram illustrates the complete workflow from user interaction to result display in the scExplorer platform. The process begins when a user selects a gene for visualization (step 1, *t* = 0s) and submits the request through the JavaScript frontend. The Node.js/EJS frontend forwards the request to the FastAPI backend (step 2), which validates data file existence (step 3) before submitting a SLURM job for processing (step 4, *t* = 0.2s). The system returns a job ID and renders a loading page with auto-refresh functionality (steps 5–6, *t* = 0.5s). The SLURM/HPC backend executes the gene visualization function using Python/R analysis modules (step 7), completing the analysis and saving results (step 8, *t* = 12s). Upon job completion, the frontend displays the interactive plot to the user (steps 9–10, *t* = 12.1s). The modular Docker-based architecture enables scalable job management through SLURM integration while maintaining responsive user interaction through asynchronous processing. Solid arrows represent synchronous requests that block until response, dashed arrows indicate asynchronous fire-and-forget requests, and green arrows show response/return flows.

The API is documented at runtime via OpenAPI (FastAPI/docs) and in a static guide within the repository. A schematic of the interaction between the frontend, backend, and SLURM scheduler is provided in Fig. 1.

#### 2.7.1 Batch correction for multi-dataset integration

Multi-sample integration represents a critical challenge in modern scRNA-seq analysis, as batch effects from sample preparation differences, sequencing protocols, or technical artefacts can mask biological variation. We address this through four complementary batch correction methods:

**ComBat** (Zhang *et al.* 2020) uses empirical Bayes frameworks to harmonize datasets from different sources, ensuring downstream clustering reflects biological rather than technical variation.

**Scanorama** (Hie *et al.* 2019) aligns disparate datasets using mutual nearest neighbours identified in reduced-dimensional space, particularly effective for large-scale integrations with significant heterogeneity.

**BBKNN** (Polański *et al.* 2020) adjusts graph-based cell relationships using local neighbourhood information from each batch, enabling detection of subtle biological variations across conditions.

**Harmony** (Korsunsky *et al.* 2019) iteratively corrects low-dimensional embeddings while preserving biological signals, making it robust for datasets with complex batch structures.

Users select and customize these methods through the web interface based on their specific integration challenges. The current web interface supports uploading and integrating up to five datasets simultaneously per run.

#### 2.7.2 Reproducibility and documentation

scExplorer generates comprehensive PDF reports documenting cell and gene counts at each workflow step, all analysis parameters, and software versions used. Configuration files can be exported in human-readable format, enabling precise replication by collaborators or reviewers. A state management system invalidates downstream results when upstream parameters are modified, displaying prominent warnings and requiring re-analysis to maintain internal consistency. Each module in the web interface includes a concise operation guide and links to a downloadable sample output illustrating expected results.

### 2.8 Extensibility and API

scExplorer exposes a documented REST API for all analysis steps (upload, preprocessing, integration, embedding, differential expression analysis, visualization, and report generation). Endpoints follow a resource-oriented design and are automatically documented via OpenAPI (FastAPI/docs). The public repository includes an API reference with request/response examples and usage notes.

### 2.9 Pre-uploaded datasets and tutorial

scExplorer comes pre-loaded with three example datasets to demonstrate its functionality, all of which are publicly available. This allows users to explore the platform without needing to upload their own data initially. The datasets are:

- **Human PBMC:** A standard benchmark dataset of 2700 peripheral blood mononuclear cells, sourced directly from 10× Genomics. This dataset is useful for familiarizing users with a typical scRNA-seq workflow.
- **Mouse Brain Cells:** A dataset of 5000 cells from a combined cortex, hippocampus, and subventricular zone of an E18 mouse, also sourced from 10× Genomics. This is useful for exploring cellular diversity in complex tissues.
- **Zebrafish Cranial Cells:** A dataset of cranial neural crest-derived cells ideal for studying developmental biology and lineage tracing (Fabian *et al.* 2022).

Additionally, scExplorer features an in-depth tutorial designed to guide users through the interface and explain each parameter in detail. The tutorial and module pages specify accepted file formats and maximum file sizes near the upload control and provide links to example datasets.

## 3 Discussion

The computational complexity of scRNA-seq analysis continues to limit its accessibility, particularly for research groups lacking dedicated bioinformatics support. While several web-based platforms have emerged to address this challenge, most focus primarily on visualization or basic analysis, leaving gaps in multi-dataset integration, scalable computation, and reproducible workflows.

Our comparative analysis reveals that existing platforms each address different aspects of the accessibility problem (see Table 1). SingleCAnalyzer (Del Toro-De León *et al.* 2022) enables analysis from raw FASTQ files but lacks robust batch correction capabilities. ICARUS (Seal *et al.* 2022) provides excellent multimodal support but cannot handle the computational demands of large-scale studies. Shaoxia (Hu *et al.* 2024) offers the broadest suite of downstream tools but provides limited options for complex batch correction scenarios.

**Table 1.** Comparative analysis of web-based scRNA-seq platforms.

Feature	scExplorer	SingleCAnalyzer (Del Toro-De León *et al.* 2022)	ICARUS (Seal *et al.* 2022)	Shaoxia (Hu *et al.* 2024)	scRNA-Explorer (Zhang *et al.* 2024)
Primary strength	Robust reproducibility, advanced batch correction, scalable job management.	Analysis from raw FASTQ files; interactive data exploration.	User-friendly tutorial interface; multimodal data support (CITE-seq).	Broadest suite of downstream tools (velocity, trajectory, SCENIC).	Specialized ‘bait’-based gene correlation analysis.
Input formats	.h5ad,.h5,.rds, 10× outputs.	FASTQ, HDF5, ENA ID.	Count matrix, RDS file, 10× outputs.	FASTQ, 10× outputs, count matrix.	Processed Seurat object.
Batch correction	Extensive suite: ComBat, Scanorama, BBKNN, Harmony.	Not explicitly mentioned.	Harmony, CCA, RPCA.	Standard Seurat methods.	Standard Seurat methods (e.g. integration anchors).
Job management	SLURM-based: Pause, queue, resume jobs with email notifications.	Not mentioned.	Not mentioned.	SLURM-based: Task scheduling on HPC.	Not mentioned.
Reproducibility	Automated PDF reports; exportable config files with all parameters and versions.	Generates reports, but lacks parameter/version export feature.	Save/Continue via ‘.Rdata’ file; logs user input parameters.	Stores parameters in a database; no mention of exportable config files.	Code on GitHub; no mention of run-specific parameter logging or session saving.

scExplorer addresses three specific limitations that we identified as critical barriers to rigorous collaborative research. First, comprehensive batch correction using multiple algorithms enables researchers to handle complex experimental designs that are increasingly common as studies integrate data from multiple laboratories, conditions, or time points. Second, SLURM-based job management makes large-scale analyses feasible on shared computational resources, removing hardware limitations that restrict many research groups. Third, automated reporting and configuration export ensure that analyses can be precisely replicated, addressing the reproducibility challenges that have hindered collaborative scRNA-seq research.

The scientific impact of these improvements extends beyond convenience. Proper batch correction enables identification of subtle biological signals that might be masked by technical variation, particularly important for studies of rare cell populations or developmental transitions. Scalable computation allows analysis of atlas-scale datasets that can reveal cell type diversity not apparent in smaller studies. Robust reproducibility supports collaborative research and enables method validation across laboratories.

However, we acknowledge current limitations that inform future development priorities. Browser-based visualization becomes less responsive with datasets exceeding 100 000–200 000 cells; to mitigate this, scExplorer automatically rasterizes large plots to preserve interactivity and, where appropriate, supports downsampling and density-based rendering. The platform does not currently process raw FASTQ files, requiring users to generate count matrices using external tools. Our focus on core analysis reliability means fewer specialized downstream modules compared to platforms like Shaoxia, though the documented API provides a path for external tools to interoperate with scExplorer.

### 3.1 Limitations and future directions

Several technical and scope limitations currently constrain scExplorer’s applicability and inform our development roadmap.

**Data Processing Scope:** scExplorer requires pre-processed count matrices and cannot analyse raw FASTQ files directly. Users must employ external tools like Cell Ranger, potentially introducing workflow fragmentation for groups seeking end-to-end solutions. Additionally, we do not currently support specialized data types such as spatial transcriptomics, CITE-seq, or ATAC-seq integration.

**Analytical Depth vs. Breadth Trade-off:** Our focus on robust core functionality means scExplorer currently offers fewer specialized downstream analyses compared to platforms like Shaoxia (Hu *et al.* 2024). Advanced methods such as RNA velocity, trajectory inference, pseudotime analysis, and gene regulatory network reconstruction are not yet integrated.

**Customization Limitations:** While visualization options are comprehensive, some users may require greater control over statistical parameters or algorithmic implementations. The web interface necessarily abstracts complex parameter spaces, which may frustrate users who need fine-grained control over analysis pipelines.

**Deployment Complexity:** Although Docker simplifies installation, configuring SLURM integration and managing multi-user deployments requires system administration expertise. This may limit adoption in resource-constrained environments or groups lacking IT support.

Near-term development focuses on improving scalability for million-cell datasets through server-side visualization aggregation and progressive data loading. Medium-term goals include support for spatial transcriptomics and tighter interoperability with popular single-cell databases for seamless data import.

### 3.2 Sustainability and community model

We maintain a public roadmap and adopt semantic versioning with tagged releases. The repository includes contribution guidelines and issue templates to facilitate community participation and transparent development. Institutional support is provided by Fundación Ciencia y Vida and Universidad San Sebastián, including infrastructure and personnel resources to ensure ongoing maintenance and availability. We welcome community feedback and contributions via the public issue tracker and documented review process.

## Data Availability

scExplorer is accessible at http://apps.cienciavida.org/scexplorer, allowing users to perform single-cell RNA sequencing (scRNA-seq) data analysis directly through a web interface without the need for local installations. For users interested in deploying their own instance or contributing to the project, the source code is open and freely available on GitHub at https://github.com/networkbiolab/scexplorer.
